# University Student's Knowledge, Practices, and Perceptions of Food Packaging Labels in Bangladesh: A Cross‐Sectional Study

**DOI:** 10.1002/fsn3.70567

**Published:** 2025-07-04

**Authors:** Nur Mohammed Imran Hossain, Md. Entaduzzaman Jony, Sohanur Rahman Emon, Naimul Islam Sifat, Md. Arshad Rahman, Md. Golam Maula Shomrat, Md. Mahbub Alam Jony

**Affiliations:** ^1^ Department of Food Engineering Gopalganj Science and Technology University Gopalganj Bangladesh

**Keywords:** behavior, food safety, labeling literacy, packaging preferences, public health, student awareness

## Abstract

Food packaging labels play a crucial role in ensuring consumer safety and aiding informed decision‐making. However, in Bangladesh, limited research exists on students' understanding and use of food packaging labels. This cross‐sectional study assessed the knowledge, practices, and consumer preferences of food labeling among 397 students from various public and private universities across Bangladesh. A self‐administered questionnaire, developed based on food safety regulations, was used for data collection. The study employed convenience sampling, and data analysis was conducted using SPSS 27.0, including descriptive statistics, Chi‐square tests, and Spearman's rho correlation to evaluate relationships between knowledge and practices. The study found that 74.8% of students had adequate knowledge of food labeling, with high awareness of “Expiry Date” and “Nutritional Information,” but limited understanding of “Batch Number” and “Packaging Symbols”. Only 44.3% demonstrated good labeling practices, with gaps in checking “Best Before” dates, allergen content, and barcodes. Gender and residential background significantly influenced knowledge and practices (*p* < 0.001), with females and urban students performing better. A significant positive correlation (*r*_*s*_ = 0.524, *p* < 0.01) indicated that higher knowledge led to better practices. Consumer preferences emphasized food protection, quality, and sustainability, with Kraft/Carton as the preferred packaging material. Although students possess sufficient knowledge, their labeling practices remain lacking, highlighting the need for targeted educational initiatives and policy measures to address this gap and encourage more informed food choices.

## Graphical abstract

Knowledge‐Practice Gap in Food Packaging Label Use Among University Students in Bangladesh.

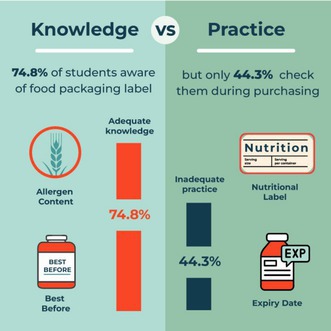

## 1 Introduction

Food labeling is a critical tool for promoting public health by enabling consumers to make informed dietary decisions, particularly in the context of rising packaged food consumption (Souza and Fernando 2016). Food packaging labels typically include essential information such as ingredients, expiry dates, and nutritional content (Patel et al. 2022). It can help to mitigate diet‐related noncommunicable diseases (NCDs), which account for over 41 million global deaths annually, with a growing burden in low‐income countries (WHO 2018). In Bangladesh, this trend is reflected in the increasing prevalence of obesity, diabetes, and cardiovascular disease, with NCDs responsible for 67% of all deaths in 2020 (WHO 2020). While regulatory bodies such as the Bangladesh Food Safety Authority (BFSA) and the Bangladesh Standards and Testing Institution (BSTI) mandate food labeling standards, enforcement is inconsistent, and many consumers, especially young adults, struggle to interpret label content effectively (Islam et al. 2024; Ali and Shahnewaj 2017).

Consumer awareness encompasses knowledge of labeling regulations, nutritional content, and product safety (Tumulak et al. 2015). According to Perumal et al. (2022), food labels are designed to help consumers make informed choices, but research suggests that many consumers do not consistently read or understand key labeling components such as barcodes, food additives, and allergen warnings (Boon and Bozinovski 2019). Studies from other developing nations have reported low engagement with food labels due to factors like literacy difficulties, misleading marketing, and unclear labeling formats (Mandle et al. 2015). Furthermore, demographic factors such as education, gender, income, and place of residence significantly influence consumers' ability to read labels and their purchasing behaviors (Patel et al. 2022; Todd et al. 2021). However, limited research has been conducted in Bangladesh to assess how university students, who represent a significant segment of future consumers, engage with food packaging labels.

Despite existing food labeling policies in Bangladesh, research on how young adults interpret and use food labels remains scarce. Most studies focus on regulatory aspects rather than actual consumer behavior (Islam et al. 2024; Taillie et al. 2024). Additionally, there is little demographic‐specific research on how factors such as education level, socioeconomic status, and dietary habits influence food labeling comprehension.

This study assessed food labeling knowledge and practices among university students in Bangladesh, while also investigating the relationship between demographic factors and labeling behavior. It also examines consumer preferences when selecting food products and their packaging to identify the key attributes that influence purchasing decisions. By analyzing these factors, the research aims to provide practical insights for policymakers, regulatory bodies, and consumer advocacy groups to improve awareness of food labeling and ensure compliance.

## 2 Methodology

### 2.1 Study Design

Using a cross‐sectional design, university students were evaluated on their knowledge and practices regarding food packaging and labeling, and attributes that influence purchasing decisions in Bangladesh. Bangladesh has a growing higher education sector that comprises 171 universities, including approximately 50 and more than 100 public and private universities, respectively, enrolling nearly 1.1 million total university students (BANBEIS 2024).

### 2.2 Sample Size and Sampling Method

To determine the required sample size, Cochran's formula (Cochran 1977) was applied, considering a total university student population of about 1.1 million (BANBEIS 2024). A 95% confidence level and a 5% margin of error were used for the estimation.

### 2.3 Cochran's Formula

$$n_{0}=\frac{\left(Z^{2}\times p\times \left(1-p\right)\right)}{e^{2}}$$

Where *n*_0_ = required sample size; *Z* = Z‐score for the desired confidence level (1.96 for 95%); *p* = estimated proportion of the population (0.5 for maximum variability); *e* = margin of error (0.05).

Calculation:

$$n_{0}=\frac{\left(1.96^{2}\times 0.5\times \left(1-0.5\right)\right)}{0.05^{2}}=384.16$$

Therefore, the minimum required sample size was approximately 384. To enhance the reliability of the study, a total of 397 students were ultimately selected.

A convenience sampling method was employed to collect data from easily accessible participants. The eligible participants included those who were 17 years of age or older and were enrolled in public or private universities in Bangladesh. They needed to be proficient in English and Bangali languages and have resided in their respective divisions for at least 1 year. To maintain the study's relevance, we excluded non‐Bangladeshi students, individuals under 17 years of age, and those who were not residents of the divisions.

### 2.4 Data Collection and Questionnaire Development

A self‐administered questionnaire was created based on previous studies and the Bangladesh Food Safety (Labeling of Packaged Food) Regulations (BFSA 2023; Mahdavi et al. 2012; Riaz et al. 2022), with modifications for relevance. A pilot test involving 50 students assessed clarity, readability, and timing. Based on the feedback, refinements were made. During the final analysis, we excluded the results from the pilot study.

**FIGURE 1:**
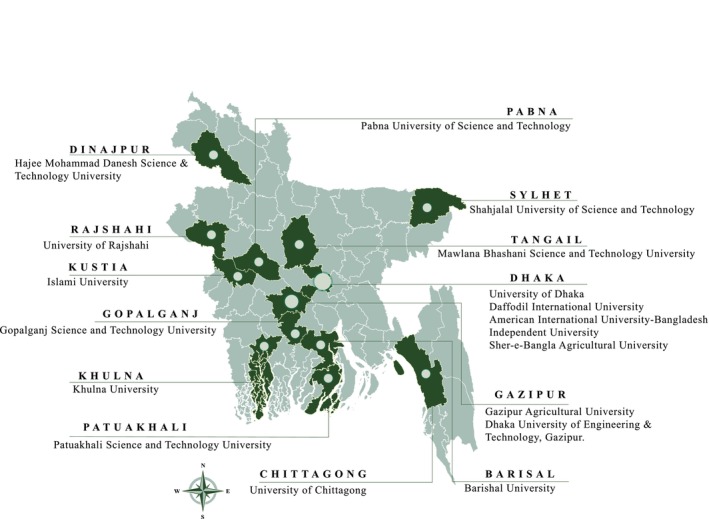
Map of sample collection sites from universities across Bangladesh.

Data collection was carried out between October and December 2024 by trained senior students specializing in food safety and food science from the Food Engineering Department at GSTU, Gopalganj. Participants were recruited from a total of 18 universities across Bangladesh, comprising both public and private universities, to ensure a diverse and representative sample (Figure 1). The universities were selected using random sampling to minimize selection bias and enhance generalizability. Within each university, students were approached in common areas such as cafeterias, libraries, and lecture halls, and invited to participate voluntarily. From each university, 18 to 26 students were recruited, depending on availability and willingness to participate, as well as having provided informed consent before completing the questionnaire.

The final questionnaire comprised 32 questions divided into four sections: demographic information, food packaging label knowledge, reading practices, and consumer perception. The demographic section collected data on marital status, religion, age, gender, geographic region (urban, suburban, rural), families' monthly income (in Bangladeshi Taka), and educational qualifications. The knowledge section assessed students' awareness of labeling elements such as “Best Before,” “Expiry Date,” “Net Quantity,” “Nutritional Information,” and “Packaging Symbols” measured by a five‐point Likert scale, where No. 1 was “No Idea,” and No. 5 was “Very Good.” Knowledge scores were categorized as poor (≤ 50%) or good (> 50%) (Agüeria et al. 2018; Imran Hossain et al. 2025). The practice section evaluated label‐reading practices through 10 questions using a five‐point scale (1 = Never, 5 = Always). Correct responses were scored from 1 (Never) to 5 (Always), with reverse scoring for incorrect practices. Scores were categorized as poor practices (≤ 50%) or good practices (> 50%) (Agüeria et al. 2018). The last section, about consumer perception of food product packaging and selection, included five questions.

### 2.5 Validity and Reliability

Construct validity was assessed using Exploratory Factor Analysis (EFA) via Principal Component Analysis (PCA) with Varimax rotation. The Kaiser–Meyer–Olkin (KMO) value was 0.843, and Bartlett's Test of Sphericity was significant (*χ* ^2^ = 3776.702, df = 45, *p* < 0.001), confirming the data's suitability for factor analysis (Kaiser 1974; Bartlett 1954). Two components were extracted (Food labeling knowledge and practice) with all item loadings exceeding 0.63, indicating strong construct validity (Field 2013). The questionnaire demonstrated excellent internal consistency, with Cronbach's alpha values of 0.940 for the knowledge section and 0.904 for the practice section (Santos 1999).

### 2.6 Ethical Considerations

Ethical approval was obtained from the Research Cell of GSTU, Gopalganj, Bangladesh. Participants were informed about the study's purpose, anonymity, and voluntary participation, with no financial incentives provided.

### 2.7 Data Analysis

An analysis of the data was performed using version 27.0 of SPSS, which stands for Statistical Package for the Social Sciences. A descriptive analysis (means, frequencies, percentages) was conducted to summarize demographic characteristics. Chi‐square tests examined associations between demographic factors and labeling behaviors, while Spearman's rho correlation assessed relationships between knowledge and practices. A significance level of 0.05 was applied to ensure statistical reliability.

## 3 Result

### 3.1 Demographic Characteristics of the University Students

**FIGURE 2:**
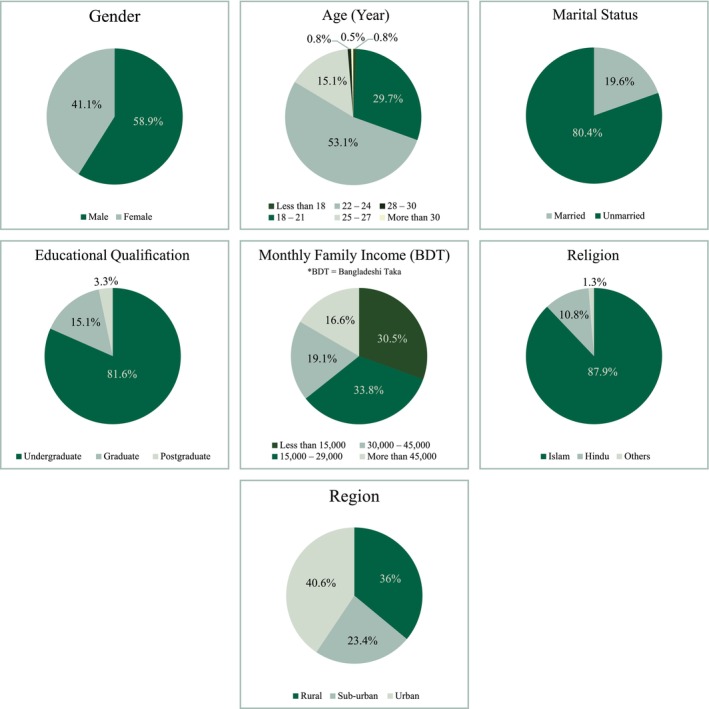
Demographic characteristics of respondents.

The respondent characteristics are demonstrated in Figure 2. Most respondents (58.9%) were males, between 22 and 24 years of age (53.1%), based on the demographic analysis. Most of them were unmarried (80.4%) and pursuing undergraduate studies (81.6%). In terms of family income, most of the respondents were found to be in the category of 15,000–29,000 BDT. Additionally, 87.9% of respondents identified as Muslim. Regionally, 40.6% lived in urban areas, 36% in rural areas, and 23.4% in suburban settings, influencing their familiarity with packaging and labeling practices.

### 3.2 Food Packaging and Labelling Knowledge

Table 1 demonstrates the level of knowledge regarding food packaging and labeling. The study found that most university students (74.8%) understand food packaging and labeling well. Students are most aware of the “Expiry Date” or “Use By Date”. They also recognize “Net Quantity,” “Nutritional Information,” and “Food Preservatives”. This shows that they are generally familiar with key details that affect their health and food choices. On the other hand, students struggle with understanding “Batch Number” or “Lot Number”. About 18.1% of students are unaware of this label, and 28.2% have only a little knowledge. Knowledge of packaging symbols and barcodes is also low, showing limited familiarity with product tracking and recycling. The understanding of food additives and allergen information is moderate, with “Food Additives” and “Allergen Content”.

**TABLE 1:** Food packaging and labeling knowledge of respondents (*N* = 397).

Statement	No idea (%)	A little (%)	Somewhat (%)	Good (%)	Very good (%)	Avg.	SD	Cronbach's Alpha
Do you know what “Best Before” means on food packaging?	36 (9.1)	50 (12.6)	234 (58.9)	58 (14.6)	19 (4.8)	2.93	0.908	0.940
Do you understand what “Expiry Date” or “Use By Date” means?	9 (2.3)	21 (5.3)	177 (44.6)	137 (34.5)	53 (13.4)	3.51	0.872	
Do you know what “Net Quantity” refers to (e.g., weight or size of the product)?	16 (4.0)	50 (12.6)	230 (57.9)	73 (18.4)	28 (7.1)	3.12	0.861	
Do you understand the purpose of “Batch Number” or “Lot Number”?	72 (18.1)	112 (28.2)	151 (38)	46 (11.6)	16 (4.0)	2.55	1.042	
Do you know why “Barcodes” are used on food packaging?	47 (11.8)	44 (21.2)	180 (45.3)	60 (15.1)	26 (6.5)	2.83	1.036	
Are you familiar with common “Packaging Symbols” (e.g., recyclable, eco‐friendly)?	45 (11.3)	110 (27.7)	185 (46.6)	35 (8.8)	22 (5.5)	2.70	0.975	
Do you understand how to read “Nutritional Information” on food labels?	20 (5.0)	58 (14.6)	226 (56.9)	67 (16.9)	26 (6.5)	3.05	0.882	
Do you know about “Food Additives” (e.g., colors, preservatives) and their labeling?	32 (8.1)	93 (23.4)	198 (49.9)	56 (14.1)	8 (4.5)	2.84	0.924	
Are you familiar with the role of “Food Preservatives”?	19 (4.8)	71 (17.9)	207 (52.1)	75 (18.9)	25 (6.3)	3.04	0.901	
Do you understand what “Allergen Content” means (e.g., peanuts, gluten)?	31 (7.8)	84 (2.2)	209 (52.6)	56 (14.1)	17 (4.3)	2.86	0.905	
Knowledge level (%)	74.8% (adequate)							

Abbreviations: Avg., average; SD, standard deviation.

### 3.3 Food Packaging and Labelling Practice

Table 2 presents an overview of university students' food packaging and labeling practices in the study. The findings indicate that university students in Bangladesh have inadequate practices concerning food packaging and labeling, with 55.7% demonstrating poor practices and only 44.3% exhibiting adequate behavior. A significant number of students do not consistently read food labels before making purchases; 21.4% never check the labels, while only 7.6% always do so. Additionally, important food safety indicators, such as “Best Before” (mean = 2.40) and “Expiry Date” (mean = 2.37), are frequently overlooked, with more than half of the students rarely or never checking these details. Practices such as checking the “Net Quantity” and “Food Additives” before purchase had slightly higher mean scores, 2.63 and 2.50, respectively. Notably, only 6% of students regularly check batch or lot numbers, indicating a lack of concern for product traceability. Furthermore, checking allergen content was limited; 28.2% of students never consider allergens before purchasing or consuming food, which poses potential health risks. The use of barcodes and awareness of packaging symbols were also low. Additionally, checking whether food is labeled as “Natural” or “Artificial” was not a common habit, as many students struggled to distinguish between naturally sourced and artificially processed food items.

**TABLE 2:** University students' practices regarding food packaging and labeling (*N* = 397).

Statement	Never (%)	Rarely (%)	Sometimes (%)	Often (%)	Always (%)	Mean	Std. Deviation	Cronbach's Alpha
Read food labels before purchasing packaged foods	85 (21.4)	108 (27.2)	142 (35.8)	32 (8.1)	30 (7.6)	2.53	1.138	0.904
Check the “Best Before”	108 (27.2)	112 (28.2)	116 (29.2)	34 (8.6)	27 (6.8)	2.40	1.169	
Check the “Expiry Date” or “Use By Date”	116 (29.2)	117 (29.5)	954 (23.7)	40 (10.1)	30 (7.6)	2.37	1.215	
Look at the “Net Quantity” (weight/volume)	75 (18.9)	106 (26.7)	138 (34.8)	45 (11.3)	33 (8.3)	2.63	1.157	
Check the “Batch Number” or “Lot Number”	124 (31.2)	114 (28.7)	101 (25.4)	34 (8.6)	24 (6.0)	2.29	1.170	
Look at the “Barcode”.	120 (30.2)	107 (27.0)	109 (27.5)	36 (9.1)	25 (6.3)	2.34	1.180	
Check “Packaging Symbols” (e.g., recyclable, biodegradable)	85 (21.4)	110 (27.7)	140 (35.3)	34 (8.6)	28 (7.1)	2.52	1.129	
Look for “Food Additives” (e.g., preservatives, food colors)	76 (19.1)	125 (31.5)	139 (35.0)	35 (8.8)	22 (5.5)	2.50	1.070	
Check the “Allergen Content” (e.g., peanuts, gluten) before buying and consumption	112 (28.2)	94 (23.7)	112 (28.2)	39 (9.8)	40 (10.1)	2.50	1.273	
Check if the food is “Natural” or “Artificial”	114 (28.7)	96 (24.2)	10 (27.2)	41 (10.3)	38 (9.6)	2.48	1.268	
Practice level	55.7% (inadequate)							

Abbreviations: Avg., Average; SD, Standard Deviation.

### 3.4 Demographic Characteristics of University Students' Knowledge and Practice

Table 3 illustrates the connections between various demographic factors and university students' understanding and food packaging and labeling practices. The results indicate that significant relationships were found with gender and residential background, while other demographic factors exhibited mixed outcomes. Gender was strongly associated with both knowledge (*χ* ^2^ = 14.243, *p* < 0.001) and practices (*χ* ^2^ = 100.184, *p* < 0.001). Females showed greater knowledge and higher rates of engagement in appropriate labeling practices compared to males. Furthermore, residential background played a critical role, as urban students reported higher levels of knowledge and practices than suburban and rural students (*p* < 0.001). Marital status also had a significant impact on practice levels (*χ* ^2^ = 19.621, *p* < 0.001), with married students demonstrating better practices compared to their unmarried peers. Additionally, monthly income was identified as a significant factor (*χ* ^2^ = 11.766, *p* = 0.008), with students earning above 45,000 BDT displaying superior labeling practices than those in lower‐income brackets.

**TABLE 3:** Association between demographic characteristics and university students' knowledge and practices on packaging and labeling (*N* = 397).

Variable	Frequency	Knowledge level				Practice level			
		Good knowledge	Poor knowledge	*χ* ^2^	*p*	Good practice	Poor practice	*χ* ^2^	*p*
*Gender*					< 0.001				< 0.001
Male	234	159	75	14.243		55	179	100.184	
Female	163	138	25			121	42		
*Age*				5.895	0.317			7.050	0.217
Less than 18	3	2	1			2	1		
19–21	118	80	38			53	65		
22–24	211	162	49			100	111		
25–27	60	49	11			18	42		
28–30	3	2	1			2	1		
More than 30	2	2	0			1	1		
*Religion*				3.735	0.155			5.118	0.077
Islam	349	266	83			158	191		
Hindu	43	27	16			14	29		
Others	5	4	1			4	1		
*Marital status*				3.741	0.053			19.621	< 0.001
Married	78	65	13			52	26		
Unmarried	319	232	87			124	195		
*Educational qualification*				2.635	0.268			0.194	0.908
Undergraduate	324	237	87			144	180		
Graduate	60	49	11			27	33		
Postgraduate	13	11	2			5	8		
*Belong*				48.653	< 0.001			40.581	< 0.001
Rural	143	80	63			37	106		
Sub‐urban	93	71	22			39	54		
Urban	161	146	15			100	61		
*Monthly income (BDT)*				3.002	0.391			11.766	0.008
Less than 15,000	121	87	34			41	80		
15,000–29,000	134	97	37			59	75		
30,000–45,000	76	59	17			37	39		
More than 45,000	66	54	12			39	27		

Abbreviation: BDT, Bangladeshi Taka.

### 3.5 Correlation Between Knowledge and Practice

Table 4 displays the connection between university students' knowledge and their practices concerning food packaging and labeling. The Spearman's rho correlation analysis reveals a significant positive correlation (*ρ* = 0.524, *p* < 0.01) between students' knowledge levels and their labeling practices. This indicates a moderate to strong association, suggesting that students who have more knowledge about food labeling are more inclined to adopt correct labeling practices. The correlation is highly significant (*p* = 0.00), indicating that this relationship is unlikely to occur by chance.

**TABLE 4:** Correlation between respondents' knowledge and practice levels (*N* = 397).

Level	Spearman's rho	Sig. (2‐tailed)
Knowledge‐practice	0.524**	0.00

** Correlation is highly significant at the *p* < 0.001 level.

### 3.6 Consumer Preferences in Food Product Selection and Packaging

Table 5 displays the results regarding consumer preferences in food product selection and packaging. The results showed that consumers primarily prioritize quality (73.3%) in food product selection, followed by price (14.1%). Packaging, brand, and convenience have less influence. In terms of packaging materials, protection of the food is the main concern (87.2%), with informative labeling and sustainability also noted. Kraft/Carton is the most preferred material, followed by glass, while plastic and metal are less favored. For food labels, best‐before dates receive the most attention (41.6%), followed by net weight and ingredient information. Certifications receive moderate attention, while the place of manufacture is the least considered factor (5.0%).

**TABLE 5:** University student's preferences for food packaging and product attributes.

Variable name	Value	Frequency	Percentage
What is the first factor that influence you while selecting a food product?	Product quality	291	73.3
	Price point	56	14.1
	Packaging design	20	5.0
	Brand reputation	16	4.0
	Simplicity	14	3.5
What should be the key characteristics of packaging's raw material?	Food protection	346	87.2
	Structural durability	8	2.0
	Flexibility of material	36	9.1
	Lightweight structure	7	1.8
What should be the essential attribute for product's packaging?	Portability (ease of transport)	69	17.4
	Food related information	126	31.7
	User‐friendliness (ease of use, shelving, and storage)	84	21.2
	Recyclability and eco‐friendly design	118	29.7
Which packaging material do you find to be the most attractive?	Glass	83	20.9
	Plastic	66	16.6
	Kraft/Carton	211	53.1
	Metal	37	9.3
Which components of the label grab your attention initially?	Net weight amount	78	19.6
	Expiry/production dates	165	41.6
	List of ingredients	66	16.6
	Manufacturing location	20	5.0
	Good manufacturing practices	68	17.1

## 4 Discussion

This study assessed university students' knowledge, practices, and consumer preferences regarding food packaging and labeling in Bangladesh, with a focus on understanding the factors that influence their behavior. The findings highlight a significant gap between students' knowledge of food labeling and their actual practices. Despite adequate awareness of basic labeling components such as expiry dates, net quantity, and nutritional information, students' engagement with these labels during food purchases remains suboptimal. Additionally, the study offers insights into how demographic factors, such as gender, residential background, and family income, influence both knowledge and practices related to food labeling.

The study found that 74.8% of university students in Bangladesh had adequate knowledge of food labeling, with a strong understanding of key components like “Expiry Dates,” “Use By Dates,” “Net Quantity,” and “Nutritional Information.” These findings align with Riaz et al. (2022) which showed similar awareness among university students. Additionally, another study conducted by Madilo et al. (2020) in Ghana reported that 85.9% of tertiary students considered expiry dates crucial. However, while students demonstrated good knowledge of basic food safety indicators, their awareness of more technical elements, such as “Batch Numbers,” “Barcodes,” and “Packaging Symbols,” was limited (mean scores: Batch Numbers = 2.55, Barcodes = 2.83, Packaging Symbols = 2.70). This pattern reflects Mahgoub et al. (2007), who found that consumers are more familiar with expiry dates but often neglect traceability elements critical to food safety. The misunderstanding of “Best Before” and “Use By” dates as food safety indicators, rather than just quality markers, is consistent with Shamim et al. (2022).

Despite the students' solid knowledge of food labeling, their actual practices were alarmingly poor. The majority of respondents (55.7%) demonstrated inadequate food labeling practices. A similar finding is reported in the study by Sega et al. (2022), which demonstrates that about 40.3% of respondents showed poor practice. Although the students were aware of the importance of food labels, their engagement with these labels when making purchasing decisions was limited, which is similar to Baig et al. (2024), who found that despite recognizing the importance of labels, many students fail to use them effectively when making food choices. A significant portion of the students (21.4%) never read food labels before purchasing, and a similar proportion failed to check essential details such as “Best Before” (27.2%) and “Expiry Dates” (29.2%), which is consistent with the study conducted by Veena Suresh et al. (2024).

Significant associations were found between key demographic factors including gender, residential background, and marital status and university students' knowledge and practices regarding food labeling. Gender was the most influential demographic factor (*p* < 0.001). Female students were found to have higher levels of both knowledge and engagement with food labels compared to their male counterparts. This aligns with research by Ikonen et al. (2020), which found that women tend to be more health‐conscious and proactive about food safety than men. This gender‐based difference may be explained by the fact that women are more likely to prioritize health and well‐being in their purchasing decisions, which includes paying closer attention to food labeling. Residential background was also associated with the students' knowledge and practices (*p* < 0.001). Urban students reported higher knowledge and practice levels compared to their rural and suburban counterparts. This finding aligns with previous studies in Nigeria and Indonesia that found urban consumers generally had higher food label reading rates compared to rural counterparts (Hajijah and Retnaningsih 2024; Nwanne Monye et al. 2020). Furthermore, marital status was found to influence food labeling practices, with married students exhibiting better practices (66.7%) compared to unmarried students (38.9%). Similarly, previous research indicates that married individuals are more likely to use nutrition labels compared to their unmarried counterparts (Cheah and Yip 2017).

Knowledge and practice exhibited a positive correlation (*ρ* = 0.524, *p* < 0.001), suggesting that students who are more knowledgeable about food labeling are more likely to engage with labels in their food purchasing behavior. While this correlation is moderate, it indicates that improving knowledge can potentially enhance label‐reading practices, though other factors such as motivation, convenience, and accessibility also play a role. Similarly, Yee et al. (2022) found that high knowledge levels did not guarantee good food label usage, and other studies have also reported no significant correlation between food label knowledge and practice (AlBlooshi et al. 2025; Nisa et al. 2024).

Regarding consumer preferences, the study found that product quality was the most significant factor influencing food product selection, followed by price. Similar studies conducted by Kaya (2016) and Rahardjo (2016) align with our findings. Packaging design and brand reputation were less influential. This reflects the findings of Azuma et al. (2019) and Dolealová et al. (2016), who observed that consumers prioritize product quality when making food purchasing decisions. In terms of packaging materials, the primary concern was food protection, followed by recyclability and eco‐friendly design, which highlights growing consumer awareness about sustainability. This preference aligns with trends in other countries where there is a shift toward sustainable packaging materials, such as Kraft/Carton and glass. These materials are seen as more environmentally friendly compared to plastic, which was less favored by students (De De Feo et al. 2022; Hallez et al. 2024; Orzan et al. 2018).

To close the knowledge‐practice gap in food labeling, educational programs and interventions should be implemented that not only enhance knowledge but also encourage consistent label‐reading practices. It is also essential to move beyond awareness‐based strategies and address the behavioral and systemic factors that hinder label use. Simplifying label design through larger fonts, standardized icons, and clearer layouts can reduce cognitive overload and improve usability, especially for young consumers like university students who often find labels overwhelming or time‐consuming (Boon and Bozinovski 2019; Koen et al. 2018). Digital literacy should also be promoted, enabling students to access real‐time nutritional and safety information through QR codes or mobile applications, thereby enhancing convenience and practical use (Battalwar and Gupta 2015). Educational efforts must target practical understanding of lesser‐known but important elements such as batch numbers and allergen information, ensuring students not only recognize labels but also apply them effectively in decision‐making. At the policy level, reforms are needed to align Bangladesh's food labeling standards with international best practices. Regulatory bodies like the Bangladesh Food Safety Authority (BFSA) and the Bangladesh Standards and Testing Institution (BSTI) should collaborate to ensure labels are both consumer‐friendly and digitally accessible. A comprehensive approach integrating education, digital innovation, user‐centered design, and regulatory reform is vital for transforming food labeling knowledge into meaningful, health‐conscious consumer behavior.

## 5 Limitations

There are few limitations to this study that need to be considered. Its cross‐sectional design makes it difficult to establish causal relationships between knowledge of food labeling and related practices. Furthermore, the use of self‐reported data might lead to biases, including social acceptance and perception bias, which might compromise the relevancy of the findings. Moreover, the use of convenience sampling restricts generalizability, as participants were selected based on accessibility rather than random selection.

To improve the rigor and comprehensiveness of future research, it is essential to incorporate random sampling, qualitative methodologies, and longitudinal designs. These techniques will allow for a more sophisticated understanding of the complicated relationships toward food labeling legislation.

## 6 Conclusion

This study reveals a significant gap in university students' knowledge and practices related to food packaging labels in Bangladesh. While students recognize key aspects such as expiry dates and nutritional information, their understanding of crucial details like batch numbers, allergen content, and packaging symbols is limited. Many do not consistently check this essential information, indicating a need for better practical application of their labeling knowledge. Consumer preferences prioritize food safety, quality, and sustainability, with a notable preference for eco‐friendly packaging materials like Kraft or carton. Gender and residential background influence labeling behaviors, with female and urban students demonstrating higher engagement. The positive correlation between knowledge and practice suggests that increasing awareness can foster more informed consumer behavior. To bridge these gaps, it is essential to implement targeted educational initiatives, create standardized labeling formats, and enhance regulatory enforcement. Collaboration among universities, policymakers, and consumer advocacy groups is crucial to incorporate food labeling literacy into academic curricula and public awareness campaigns. By empowering students to effectively interpret and utilize labeling information, we can promote informed food choices and ultimately improve public health and consumer safety in Bangladesh.

## Author Contributions

**Nur Mohammed Imran Hossain:** conceptualization (lead), data curation (equal), formal analysis (equal), methodology (equal). **Md. Entaduzzaman Jony:** conceptualization (equal), resources (equal), supervision (lead), validation (equal). **Sohanur Rahman Emon:** data curation (equal), formal analysis (equal), investigation (equal), writing – original draft (lead). **Naimul Islam Sifat:** formal analysis (equal), investigation (lead), software (equal), writing – original draft (equal). **Md. Arshad Rahman:** data curation (equal), formal analysis (equal), methodology (equal), software (lead). **Md. Golam Maula Shomrat:** formal analysis (equal), project administration (equal), resources (lead), visualization (equal). **Md. Mahbub Alam Jony:** data curation (equal), formal analysis (lead), investigation (equal), writing – original draft (equal).

## Conflicts of Interest

The authors declare no conflicts of interest.

## Data Availability

Upon request, data will be provided.

## References

[fsn370567-bib-0001] Agüeria, D. A. , C. Terni , V. M. Baldovino , and D. Civit . 2018. “Food Safety Knowledge, Practices and Attitudes of Fishery Workers in Mar Del Plata, Argentina.” Food Control 91: 5–11.

[fsn370567-bib-0002] AlBlooshi, S. , L. Smail , A. Aldayyani , F. Zeb , and A. Ibrahim . 2025. “University Students' Understanding and Utilization of Food Labels: A Cross‐Sectional Study.” International Journal of Food Science 2025: 7391826. 40046253 10.1155/ijfo/7391826 PMC11882323

[fsn370567-bib-0003] Ali, A. N. M. A , and Shahnewaj . 2017. “Improper Labelling of Manufacturing and Expiry Dates of Food: A Legal and Regulatory Study of Food Quality and Food Waste in Bangladesh.” Australian Journal of Asian Law 18, no. 1: 27–40.

[fsn370567-bib-0005] Azuma, T. M. , B. N. A. Quao , and G. O. Adwoa Christabel . 2019. “Food Purchase Decisions: The Influence of Food Quality and Safety.” IJAMR 3, no. 7: 33–40.

[fsn370567-bib-0006] Baig, A. , J. S. Koippallil , S. K. J. Pallavi Gautam , et al. 2024. “IJCM_282A: Knowledge, Attitude and Practices Towards Food Labelling and Its Use Among Health Care Students of Mangalore.” Indian Journal of Community Medicine 49, no. Suppl 1: S82.

[fsn370567-bib-0007] BANBEIS . 2024. “Bangladesh Education Statistics 2023.” https://banbeis.portal.gov.bd/sites/default/files/files/banbeis.portal.gov.bd/npfblock/Bangladesh%20Education%20Statistics%202023%20(1).pdf.

[fsn370567-bib-0008] Bartlett, M. S. 1954. “A Note on the Multiplying Factors for Various Chi Square Approximations.” Journal of the Royal Statistical Society: Series B: Methodological 16, no. 2: 296–298.

[fsn370567-bib-0009] Battalwar, R. , and R. Gupta . 2015. “Knowledge, Attitude and Frequency of Reading Food Labels of Males and Females in Mumbai City.” Agricultural and Food Science 2, no. 3: 59018898.

[fsn370567-bib-0010] BFSA . 2023. “Food Safety (Labeling of Packaged Food) Regulations, 2023.” https://bfsa.portal.gov.bd/sites/default/files/files/bfsa.portal.gov.bd/page/c6eaf0ee_f70a_42b9_ab35_48f1ccc4878e/2023‐07‐26‐10‐19‐93d048e60100e7dfc40ee1ce0004fade.pdf.

[fsn370567-bib-0012] Boon, H. , and N. Bozinovski . 2019. “A Systematic Narrative Review of the Evidence for Labeling of Natural Health Products and Dietary Supplements.” Journal of Alternative and Complementary Medicine 25, no. 8: 777–788. 31013437 10.1089/acm.2018.0533

[fsn370567-bib-0014] Cheah, Y. K. , and C. Y. Yip . 2017. “Factors Determining the Use of Nutrition Labels: The Case of Malaysia.” Journal of Foodservice Business Research 20, no. 5: 557–567.

[fsn370567-bib-0052] Cochran, W. G. 1977. Sampling Techniques(3rd ed.). John Wiley & Sons.

[fsn370567-bib-0016] De Feo, G. , C. Ferrara , and F. Minichini . 2022. “Comparison Between the Perceived and Actual Environmental Sustainability of Beverage Packagings in Glass, Plastic, and Aluminium.” Journal of Cleaner Production 333: 130158.

[fsn370567-bib-0017] Dolealová, H. , K. Pícha , J. Navrátil , M. Veselá , and R. vec . 2016. “Perception of Quality in Decision Making Regarding Purchase of Organic Food.” Food Safety Management 7, no. 153: 86–91.

[fsn370567-bib-0018] Field, A. 2013. Discovering Statistics Using IBM SPSS Statistics. 4th ed. Sage Publications.

[fsn370567-bib-0019] Hajijah, R. N. , and R. Retnaningsih . 2024. “The the Influence of Knowledge and Risk Perception on Food Label Reading Behavior Among Adolescents in Rural and Urban Areas of Bogor.” Journal of Consumer Sciences 9, no. 1: 82–101.

[fsn370567-bib-0020] Hallez, L. , B. Spruyt , F. Boen , and T. Smits . 2024. “How Consumers Value Sustainable Packaging: An Experimental Test Combining Packaging Material, Claim and Price.” British Food Journal 126, no. 9: 3566–3583.

[fsn370567-bib-0021] Hossain, I. , N. Mohammed , M. E. Jony , et al. 2025. “Food Safety Knowledge, Attitude, and Hygiene Practices (KAP) and Microbial Quality of Meat in Slaughterhouses: A Study in Gopalganj, Bangladesh.” Journal of Agriculture and Food Research 21: 101914.

[fsn370567-bib-0022] Ikonen, I. , F. Sotgiu , A. Aydinli , and P. W. J. Verlegh . 2020. “Consumer Effects of Front‐Of‐Package Nutrition Labeling: An Interdisciplinary Meta‐Analysis.” Journal of the Academy of Marketing Science 48, no. 3: 360–383.

[fsn370567-bib-0023] Islam, M. N. , N. Roy , F. K. Madilo , et al. 2024. “Knowledge, Perception, and Practical Understanding of Food Labels: A Cross‐Sectional Study Among Bangladeshi Consumers.” Food Science & Nutrition 12, no. 10: 7552–7567. 39479638 10.1002/fsn3.4366 PMC11521752

[fsn370567-bib-0024] Kaiser, H. F. 1974. “An Index of Factorial Simplicity.” Psychometrika 39, no. 1: 31–36.

[fsn370567-bib-0025] Kaya, I. H. 2016. “Motivation Factors of Consumers' Food Choice.” Food and Nutrition Sciences 7, no. 3: 149–154.

[fsn370567-bib-0026] Koen, N. , E. Wentzel‐Viljoen , D. Nel , and R. Blaauw . 2018. “Consumer Knowledge and Use of Food and Nutrition Labelling in South Africa: A Cross‐Sectional Descriptive Study.” International Journal of Consumer Studies 42, no. 3: 335–346.

[fsn370567-bib-0027] Madilo, F. K. , J. Owusu‐Kwarteng , A. Parry‐Hanson Kunadu , and K. Tano‐Debrah . 2020. “Self‐Reported Use and Understanding of Food Label Information Among Tertiary Education Students in Ghana.” Food Control 108: 106841.

[fsn370567-bib-0028] Mahdavi, A. M. , P. Abdolahi , and R. Mahdavi . 2012. “Knowledge, Attitude and Practice Between Medical and Non‐Medical Sciences Students About Food Labeling.” Health Promotion Perspective 2, no. 2: 173–179. 10.5681/hpp.2012.020 PMC3963625 24688931

[fsn370567-bib-0029] Mahgoub, S. , P. P. Lesoli , and K. Gobotswang . 2007. “Awareness and Use of Nutrition Information on Food Packages Among Consumers in Maseru (Lesotho).” African Journal of Food, Agriculture, Nutrition and Development 7, no. 6: 1–16.

[fsn370567-bib-0030] Mandle, J. , A. Tugendhaft , J. Michalow , and K. Hofman . 2015. “Nutrition Labelling: A Review of Research on Consumer and Industry Response in the Global South.” Global Health Action 8, no. 1: 25912. 25623608 10.3402/gha.v8.25912 PMC4306755

[fsn370567-bib-0032] Monye, N. F. , N. N. Ezumah , J. Ani , H. Umezuruike , F. O. Ukwueze , and E. L. Okiche . 2020. “Examining Knowledge, Attitude and Practice Towards Food Labels Among Consumers in Enugu State, Nigeria – A Baseline Survey.” International Journal of Law and Society 3, no. 4: 221.

[fsn370567-bib-0033] Nisa, L. , N. Nuryanto , R. Purwanti , and F. F. Dieny . 2024. “Hubungan Pengetahuan Dan Sikap Terkait Label Pangan Dengan Kepatuhan Membaca Label Pangan Pada Mahasiswa Universitas Diponegoro.” Journal of Nutrition College 13, no. 1: 81–88.

[fsn370567-bib-0034] Orzan, G. , A. Cruceru , C. Bălăceanu , and R.‐G. Chivu . 2018. “Consumers' Behavior Concerning Sustainable Packaging: An Exploratory Study on Romanian Consumers.” Sustainability 10, no. 6: 1787.

[fsn370567-bib-0035] Patel, Y. K. , S. Tiwari , and A. Vithalkar . 2022. “Food Labelling: An Important Aspect of Marketing.” International Journal of Research Publication and Reviews 3, no. 11: 3230–3238.

[fsn370567-bib-0036] Perumal, K. , B. Balakrishnan , and M. Z. Idris . 2022. “Food Labelling From Consumers' Perspectives: A Review.” Muallim Journal of Social Science and Humanities 6, no. 3: 80–88.

[fsn370567-bib-0037] Rahardjo, C. R. 2016. “Faktor Yang Menjadi Preferensi Konsumen Dalam Membeli Produk Frozen Food.” Performa 1, no. 1: 32–43.

[fsn370567-bib-0038] Riaz, F. , A. Moiz , S. E. Mahmood , A. Ahmad , S. S. Abullais , and S. U. Khateeb . 2022. “Assessment of Knowledge, Attitude and Practice of Food Labeling and Expiry Date Among the Female Health Sciences Students: A Public Health Concern.” Sustainability 14, no. 11: 6708.

[fsn370567-bib-0040] Santos, J. R. A. 1999. “Cronbach's Alpha: A Tool for Assessing the Reliability of Scales.” Journal of Extension 37, no. 2: 1–4.

[fsn370567-bib-0041] Sega, J. S. , C. O. Lada , and D. T. Manafe . 2022. “The Correlation of Knowledge With Attitude and Behavior of Reading Nutrition Facts Label of Packaged Foods on Students Universitas Nusa Cendana.” EAS Journal of Nutrition and Food Sciences 4, no. 2: 28–33.

[fsn370567-bib-0042] Shamim, K. , S. Ahmad , and M. A. Alam . 2022. “Consumer Understanding of Food Date Labels: Preventing Food Wastage.” British Food Journal 124, no. 10: 3116–3132.

[fsn370567-bib-0051] Souza, V. G. L. , and A. L. Fernando . 2016. “Nanoparticles in Food Packaging: Biodegradability and Potentialmigration to Food—A Review.” Food Packaging and Shelf Life 8: 63–70.

[fsn370567-bib-0044] Taillie, L. S. , A. K. Abrar , U. Afroza , et al. 2024. “Designing Front‐Of‐Package Labels to Inform Consumers and Encourage Healthier Food Choices in Bangladesh: A Qualitative Study.” Nutrients 16, no. 23: 3989. 39683383 10.3390/nu16233989 PMC11642975

[fsn370567-bib-0045] Todd, M. , T. Guetterman , G. Sigge , and E. Joubert . 2021. “Multi‐Stakeholder Perspectives on Food Labeling and Health Claims: Qualitative Insights From South Africa.” Appetite 167: 105606. 34298013 10.1016/j.appet.2021.105606

[fsn370567-bib-0046] Tumulak, J. M. O. , J. B. Patosa , and Z. P. Ibañez . 2015. “Consumer Awareness on Labelled Food Products in Digos City.” In Joint International Conference on Agribusiness and Cooperatives, Philippines, 1–10. Oxford Research Encyclopedia of Communication.

[fsn370567-bib-0047] Veena Suresh, H. , M. Veronika , and S. Ramalingam . 2024. “Knowledge, Attitude and Practice of Reading Food Labels Among Students of Health Profession Education.” Indian Journal of Community Health 36, no. 6: 864–867.

[fsn370567-bib-0048] WHO . 2018. “Noncommunicable Diseases Country Profiles 2018.” https://www.who.int/publications/i/item/9789241514620.

[fsn370567-bib-0049] WHO . 2020. “Noncommunicable Diseases Progress Monitor 2020.” https://www.who.int/publications/i/item/ncd‐progress‐monitor‐2020.

[fsn370567-bib-0050] Yee, S. , A. W. M. N. Lloo , N. E. F. Rosle , et al. 2022. “Association Between Knowledge, Attitude, and Practice of Nutrition and Food Labels Among Selected Higher Educational Institution Students in Klang Valley.” Jurnal Sains Kesihatan Malaysia 20, no. 2: 77–85.

